# Age-Related Changes in Resting-State EEG Activity in Attention Deficit/Hyperactivity Disorder: A Cross-Sectional Study

**DOI:** 10.3389/fnhum.2017.00285

**Published:** 2017-05-30

**Authors:** Katarzyna Giertuga, Marta Z. Zakrzewska, Maksymilian Bielecki, Ewa Racicka-Pawlukiewicz, Malgorzata Kossut, Anita Cybulska-Klosowicz

**Affiliations:** ^1^Laboratory of Neuroplasticity, Department of Molecular and Cellular Neurobiology, Nencki Institute of Experimental Biology of Polish Academy of SciencesWarsaw, Poland; ^2^Gösta Ekman Laboratory, Department of Psychology, Stockholm UniversityStockholm, Sweden; ^3^Department of Psychology, SWPS University of Social Sciences and HumanitiesWarsaw, Poland; ^4^Department of Child Psychiatry, Medical University of WarsawWarsaw, Poland

**Keywords:** attention deficit/hyperactivity disorder, electroencephalography, resting-state, linear regression, development, ADHD, EEG

## Abstract

Numerous studies indicate that attention deficit/hyperactivity disorder (ADHD) is related to some developmental trends, as its symptoms change widely over time. Nevertheless, the etiology of this phenomenon remains ambiguous. There is a disagreement whether ADHD is related to deviations in brain development or to a delay in brain maturation. The model of deviated brain development suggests that the ADHD brain matures in a fundamentally different way, and does not reach normal maturity at any developmental stage. On the contrary, the delayed brain maturation model assumes that the ADHD brain indeed matures in a different, delayed way in comparison to healthy age-matched controls, yet eventually reaches proper maturation. We investigated age-related changes in resting-state EEG activity to find evidence to support one of the alternative models. A total of 141 children and teenagers participated in the study; 67 diagnosed with ADHD and 74 healthy controls. The absolute power of delta, theta, alpha, and beta frequency bands was analyzed. We observed a significant developmental pattern of decreasing absolute EEG power in both groups. Nonetheless, ADHD was characterized by consistently lower absolute EGG power, mostly in the theta frequency band, in comparison to healthy controls. Our results are in line with the deviant brain maturation theory of ADHD, as the observed effects of age-related changes in EEG power are parallel but different in the two groups.

## Introduction

Attention deficit/hyperactivity disorder (ADHD) is one of the most commonly diagnosed neurodevelopmental disorders, with reported prevalence rates of 3–7% in school-aged children (Scahill and Schwab-Stone, 2000; Burd et al., 2003; Polanczyk and Jensen, 2008). ADHD is characterized by a pattern of developmentally inappropriate levels of inattention, impulsivity, and overactivity (American Psychiatric Association, 2000). Moreover, it is frequently related to severe co-morbidities such as oppositional defiant disorder (ODD) or conduct disorder (CD), poor academic performance, and socioeconomic problems (Biederman et al., 2006).

A growing body of research on the causes of behavioral symptoms in ADHD provides evidence for impairments on genetic, neurotransmission, neuroanatomic and functional levels (for review see, Kuntsi et al., 2006), the etiology still remains unknown. As the deficits observed in ADHD may be diverse, it is regarded as a heterogeneous, multidimensional disorder, and may have multiple causes. One of the most prominent hypotheses states that ADHD results from altered brain maturation. However, there is a disagreement whether ADHD is related to a delay in brain maturation (Mann et al., 1992; Rubia, 2007; Shaw et al., 2007) or whether ADHD’s brain maturation process represents complete deviation from the typical development (Chabot and Serfontein, 1996; Dickstein et al., 2006).

Delayed brain maturation is mainly indicated by the results from structural brain imaging studies. Two longitudinal studies investigating large samples of children demonstrated that ADHD individuals follow a similar to normal sequential pattern of cortical thickening. However, it is delayed for about 2–3 years depending on the specific cortex region (Shaw et al., 2007, 2012), with largest delays observed in the prefrontal cortex.

Conversely, the findings from electroencephalographic (EEG) studies indicate persistent abnormalities in ADHDs’ EEG activity (for review see, Barry et al., 2003). For example, Chabot and Serfontein (1996) reported increased absolute and relative theta activity in frontal and frontal midline regions. Other research reported decreased relative alpha activity over parietal and temporal sites (Bresnahan and Barry, 2002; Poil et al., 2014), decreased absolute and relative beta activity in frontal, parietal, and temporal sites (Hobbs et al., 2007), and elevated theta/beta (TBR) and theta/alpha ratios (TAR) (Hermens et al., 2005).

Elevated theta power and TBR are the most frequently reported EEG abnormalities in ADHD and have been interpreted as indices of immature brain activity. Some researchers proposed the aforementioned indices as a biomarker of ADHD (Magee et al., 2005; Snyder and Hall, 2006). However, they did not obtain clinical acceptance and use (Lenartowicz and Loo, 2014; Loo et al., 2016). Moreover, recent studies failed to replicate the effect of elevated theta and TBR in ADHD (Loo et al., 2009, 2013; van Dongen-Boomsma et al., 2010; Liechti et al., 2013). A meta-analysis revealed that TBR effect size is negatively related to the year of publication, methodological factors, inclusion and exclusion criteria, and a decreasing trend in sleep duration across time were proposed as possible explanations for this observation (Arns et al., 2013).

Interestingly, despite the importance of age-related effects for the understanding of ADHD, all of the mentioned EEG results come from quasi-experimental studies that contrasted the EEG indices of younger and older participants. To the best of our knowledge, there are no longitudinal or cross-sectional studies focused on tracking developmental trends in EEG activity using a regression approach. A study by Clarke et al. (2001) investigated differences in two subtypes of ADHD. They reported that the differences in power between ADHD inattentive and control groups remained constant with increasing age (from 8 to 12 years old). However, power in the ADHD combined group changed at a greater rate than in the ADHD inattentive group, with power of the two ADHD groups becoming similar with age. Additionally, sex differences between the ADHD and control subjects in all EEG absolute power measures were more pronounced in males and matured faster in males in comparison to females.

The developmental trends of EEG activity in healthy participants have been reported widely for decades (Matousek and Petersen, 1973; Barriga-Paulino et al., 2011). The index of this trend is a decrease in the power of all frequency bands, with the most prominent decrease in low frequencies: delta and theta (for a review see, Segalowitz et al., 2010; Uhlhaas et al., 2010). Maturational EEG power decrease is observed globally over all scalp sites (Whitford et al., 2007). The exact physiological process underpinning this effect remains unclear. A few recent studies tried to address this problem using simultaneous EEG recordings and MRI scanning. One of them observed that the age-related decrease of EEG power was correlated with a decline in gray matter density (Whitford et al., 2007), which has been interpreted as a reduction of neuropil and elimination of active synapses. The latter may be responsible for the age-related EEG power reduction.

Both imaging evidence on the delayed cortical maturation in ADHD and the well-established age-related changes in EEG activity in healthy participants inspired our study. The main goal of the project was to study the extent to which developmental pattern of resting EEG activity is similar in ADHD and typical development. For that purpose, we investigated a large sample (141 participants) with a broad age range (9–16 years), allowing us to consider age as a continuous predictor in linear regression models. Linear regression analysis has been previously used to detect EEG developmental tendencies (Wackermann and Matousek, 1998), however, not in the context of ADHD and therefore we expect this analysis to reveal different developmental patterns in EEG between ADHD and healthy participants.

## Materials and Methods

A total of 150 children and teenagers (Caucasian) aged 9–16 participated in the study. Due to technical issues (five ADHD participants) and excessive motion artifacts (two ADHD participants, two controls), we included 141 participants in the final analysis. The clinical group consisted of 67 participants with an ADHD diagnosis (*M*_age_ = 13.11 ± 2.06; 12 females). The healthy control group was age- and sex- matched to the clinical group (*n* = 74, *M*_age_ = 13.17 ± 2.2; 12 females).

The clinical group was recruited among ADHD outpatients under the supervision of the Psychiatry Clinic at Public Pediatric Teaching Hospital in Warsaw, Poland. The diagnosis was performed according to diagnostic criteria of the DSM-IV TR (4th edition, text revision; American Psychiatric Association, 2000) for ADHD subtypes: predominantly inattentive, impulsive/hyperactive, and combined. The diagnostic process was conducted at the clinic by a trained and experienced team of child psychiatrists and psychologists as previously described (Hanć et al., 2014; Racicka et al., 2015), and included: an interview with patients’ parents, Diagnostic Structured Interview for ADHD and Hyperkinetic Disorder according to ICD-10 and DSM-IV TR (Wolańczyk and Kołakowski, 2005), the Behavioral Disorders Supplement of Diagnostic Interview Kiddie-SADS-Present and Lifetime Version (Kiddie-SADS-PL), and observation of patients’ behavior. Patients’ parents provided background records and school reports. Both parents and teachers completed the behavior rating scales (Wolańczyk and Kołakowski, 2005). ADHD symptoms intensity was rated using ADHD Rating Scale (ADHD-RS, DuPaul et al., 1998). The clinical group exhibit following symptoms intensity as measured with ADHD-RS: total score (*M* = 38.47, *SD* = 9.74), inattention (*M* = 21.08, *SD* = 3.82), hyperactivity (*M* = 8.98, *SD* = 4.42), impulsivity (*M* = 8.42, *SD* = 3.76). The comorbidity diagnosis was based on the diagnostic criteria for ICD-10 (World Health Organization, 1994), and the diagnosis was verified during no less than three appointments. If the results of all diagnostic methods were consistent, the diagnosis was confirmed, and such patients were invited to take part in our study. The ADHD group consisted of children diagnosed with combined (*n* = 47) or predominantly inattentive ADHD subtypes (*n* = 20). The comorbidity distribution was as follows: 66% of participants manifested comorbidities, 47% of which were diagnosed with ODD/ODC+CD, and 25.7% had learning difficulties.

The inclusion criteria for our ADHD group were: a confirmed diagnosis, no previous head injuries with a loss of consciousness, and a lack of somatic disorders and epilepsy. ADHD participants were asked to abstain from taking stimulant medication at least 24 h before testing.

A healthy control group was recruited from among Warsaw’s primary and secondary schools through advertisement at school-meetings with parents. During such a meeting the child’s parents, if interested in participating in the study, completed a questionnaire providing detailed information about their child’s health condition. It was used to select participants who did not report any attentional problems, neurological or psychiatric diagnosis, dyslexia/dysorthography, previous brain injury with the loss of consciousness, or close family members (parents, siblings) with ADHD/ADD diagnosis.

The study was approved by the local Ethics Committee at the SWPS University of Social Sciences and Humanities and the Medical University of Warsaw. All participants provided assent, and parents gave informed written consent in accordance with the Declaration of Helsinki.

### EEG Recording and Preprocessing

Resting-state EEG was recorded using a 64-channel EGI HydroCel Geodesic System (Eugen, OR, United States). The signal was sampled at 250 Hz and referenced to the Cz electrode. Electrode impedances were kept below 20 kΩ. We recorded 5 min of resting-state EEG with eyes closed (3 min) and open (2 min), alternating between the two with 1 min long sequences. The eyes open condition was introduced to prevent participants from drowsing. Following analyses were performed on signal registered while the participants had their eyes closed only. EEG data was processed offline using EEGlab software (Delorme and Makeig, 2004) and custom MATLAB scripts.

We created two datasets for each EEG file. Both datasets were 45 Hz low pass and 1 Hz high pass filtered using a Hamming windowed sinc FIR filter (transition bandwidth: 1 Hz) implemented in EEGLAB’s *pop_eegfiltnew* function. One dataset was then divided into 1-s epochs prior to independent component analysis (ICA) and visually inspected for artifacts. We removed epochs with large irregular events. Noisy electrodes (large visible drifts or net noise) were interpolated. Such short (1 s) epochs allowed for keeping more data to perform the ICA on, but were not ideal for our planned spectral analysis. Therefore, we ran the ICA on the first dataset and subsequently applied the obtained ICA weights to the second dataset, which we later epoched into 3 s long epochs. We used the *runica* algorithm as implemented in the *eeglab pop_runica* function. We identified ICA components related to eye blinks and horizontal eye movements by visual inspection, based on their scalp topography, power spectra, time course, and variance among trials (Hipp and Siegel, 2013). These eye components were removed from the signal (4.1 ± 1.84 on average). Next, we re-referenced the data to an average reference and visually inspected the datasets for any remaining artifacts, removing contaminated epochs. The average number of remaining epochs per participant was 50 ± 9.3 (out of 60). All the steps mentioned above were done using a custom MATLAB toolbox (in prep) and EEGLAB functions.

### EEG Data Analysis

We calculated a mean power spectrum for every participant using EEGLAB’s *pop*_*spectopo* function. Absolute EEG power was log transformed in order to normalize the distribution of the data for statistical analysis. All further analyses were conducted using RStudio Team (2015).

**FIGURE 1:**
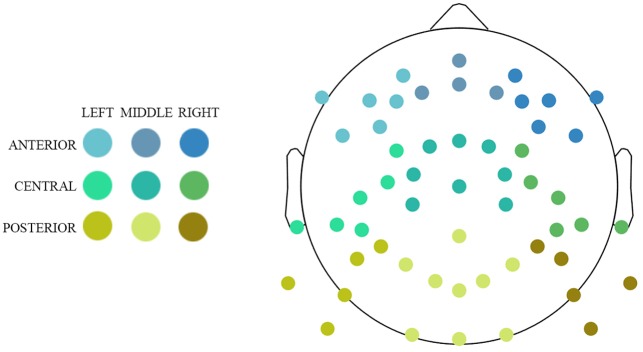
Cluster division and location of electrodes used for analysis.

We divided our data into regions of interests with regard to anterior–posterior and left–right localization on the scalp. This division resulted in nine clusters (**Figure 1**): three anteriori (left, middle, right), three central (left, middle, right), and three posterior (left, middle, right). Each participant’s power estimates were averaged across electrodes within each cluster and across frequencies within the following four frequency bands: delta (1–3 Hz), theta (3–8 Hz), alpha (8–12 Hz), and beta (12–30 Hz). Before computing the aggregates, values differing more than 2.24 median absolute deviations from the median were removed (Wilcox and Keselman, 2003).

#### Age, ADHD, and EEG Power

To test the combined effect of age and ADHD diagnosis on EEG power spectrum, we performed a regression analysis using R’s *lm()* function. Regression analyses were performed for each cluster and frequency band and included age, ADHD diagnosis, and their interaction. To explore the potential moderating role of the symptoms intensity we estimated a series of regression models in the ADHD group including ADHD-RS scores (total score or one of the three subscale scores) as moderators of age effects. To correct for multiple comparisons, we employed a false discovery rate (FDR) (Benjamini and Hochberg, 1995) within each frequency band using R’s *p.adjust* function (RStudio Team, 2015) with the *p* < 0.05 threshold.

## Results

None of the regression models estimated for all participants revealed a significant interaction effect of Age and ADHD diagnosis (all *p*-values for the interaction term > 0.14, all Δ*R*^2^ < 1.3%). Therefore, we decided to report detailed results of the simpler models, including only main effects of age and ADHD (**Table 1**). As the effects of both predictors were additive, we discuss them in turn.

**Table 1:** Linear regression analysis for the resting EEG predicted by age and diagnosis.

Frequency band/cluster	Beta (age)	*t* (age)	*p* (age)	Beta (diagnosis)	*t* (diagnosis)	*p* (diagnosis)	*R*^2^	*F* (model)
**Delta**								
Frontal left	-0.36	-3.96	**0.0001**	-0.56	-1.43	0.154	0.113	**8.79^∗∗∗^**
Frontal middle	-0.42	-5.06	**0.0000**	-0.23	-0.66	0.512	0.158	**12.98^∗∗∗^**
Frontal right	-0.40	-4.71	**0.0000**	-0.46	-1.26	0.210	0.146	**11.81^∗∗∗^**
Central left	-0.54	-6.05	**0.0000**	-0.69	-1.83	0.069	0.223	**19.82^∗∗∗^**
Central middle	-0.55	-7.08	**0.0000**	-0.28	-0.85	0.395	0.269	**25.38^∗∗∗^**
Central right	-0.56	-6.11	**0.0000**	-0.85	-2.20	0.030	0.233	**20.93^∗∗∗^**
Posterior left	-0.54	-5.52	**0.0000**	-0.89	-2.16	0.033	0.201	**17.41^∗∗∗^**
Posterior middle	-0.63	-6.68	**0.0000**	-0.96	-2.38	0.019	0.266	**24.99^∗∗∗^**
Posterior right	-0.54	-5.11	**0.0000**	-1.07	-2.39	0.018	0.186	**15.76^∗∗∗^**
**Theta**								
Frontal left	-0.37	-3.59	**0.0005**	-0.84	-1.94	0.055	0.106	**8.22^∗∗∗^**
Frontal middle	-0.35	-3.49	**0.0007**	-0.52	-1.23	0.222	0.089	**6.78^∗∗^**
Frontal right	-0.41	-4.12	**0.0001**	-0.77	-1.84	0.069	0.128	**10.10^∗∗∗^**
Central left	-0.45	-4.30	**0.0000**	-1.02	-2.32	**0.022**	0.146	**11.82^∗∗∗^**
Central middle	-0.42	-4.38	**0.0000**	-0.58	-1.42	0.158	0.132	**10.51^∗∗∗^**
Central right	-0.49	-4.56	**0.0000**	-1.14	-2.50	**0.014**	0.162	**13.36^∗∗∗^**
Posterior left	-0.41	-3.65	**0.0004**	-1.19	-2.49	**0.014**	0.123	**9.64^∗∗∗^**
Posterior middle	-0.54	-4.50	**0.0000**	-1.32	-2.59	**0.011**	0.162	**13.34^∗∗∗^**
Posterior right	-0.44	-3.56	**0.0005**	-1.62	-3.10	**0.002**	0.138	**11.01^∗∗∗^**
**Alpha**								
Frontal left	-0.03	-0.23	0.8167	-1.20	-1.92	0.057	0.026	1.87
Frontal middle	0.12	0.78	0.4380	-1.10	-1.72	0.088	0.025	1.79
Frontal right	-0.08	-0.56	0.5750	-1.33	-2.19	0.030	0.036	2.54
Central left	-0.22	-1.60	0.1129	-1.38	-2.36	0.020	0.055	4.01^∗^
Central middle	-0.09	-0.60	0.5468	-1.06	-1.70	0.092	0.023	1.61
Central right	-0.24	-1.66	0.0999	-1.65	-2.71	**0.008**	0.067	**4.98^∗∗^**
Posterior left	-0.12	-0.79	0.4308	-1.39	-2.07	0.040	0.034	2.44
Posterior middle	-0.18	-0.98	0.3300	-1.25	-1.62	0.109	0.025	1.76
Posterior right	-0.17	-1.03	0.3058	-1.89	-2.65	**0.009**	0.055	4.01^∗^
**Beta**								
Frontal left	-0.26	-2.69	**0.0081**	-0.49	-1.20	0.233	0.059	**4.29^∗∗^**
Frontal middle	-0.22	-2.46	**0.0150**	-0.31	-0.81	0.417	0.046	**3.34^∗^**
Frontal right	-0.28	-2.84	**0.0051**	-0.31	-0.73	0.465	0.058	**4.29^∗^**
Central left	-0.28	-2.99	**0.0033**	-0.49	-1.22	0.224	0.070	**5.16^∗∗^**
Central middle	-0.16	-1.62	0.1082	-0.42	-1.03	0.304	0.026	1.82
Central right	-0.33	-3.36	**0.0010**	-0.89	-2.12	0.036	0.102	**7.81^∗∗∗^**
Posterior left	-0.19	-1.93	0.0556	-0.57	-1.39	0.168	0.039	2.79
Posterior middle	-0.12	-1.20	0.2330	-0.19	-0.47	0.640	0.012	0.82
Posterior right	-0.28	-2.82	**0.0055**	-0.86	-2.05	0.042	0.080	**6.02^∗∗^**


*Model: *power ∼ age+diagnosis. ^∗∗∗^p* < 0.001; ^∗∗^*p* < 0.01; ^∗^*p* < 0.05; *p* < 0.05 after FDR correction indicated in bold.*

**FIGURE 2:**
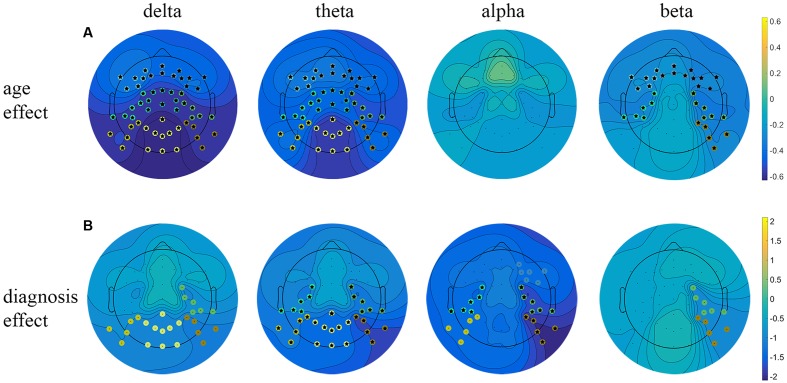
Linear regression analysis of the resting EEG predicted by age and diagnosis. Beta coefficient maps for two main effects (age and diagnosis) are plotted respectively for all frequency bands. **(A)** Represents beta coefficients for age main effect, **(B)** represents beta coefficients for diagnosis effect. Significant clusters (*p* < 0.05, uncorrected) are marked with dots in cluster-corresponding colors. Star signs indicate significant clusters after FDR correction.

**FIGURE 3:**
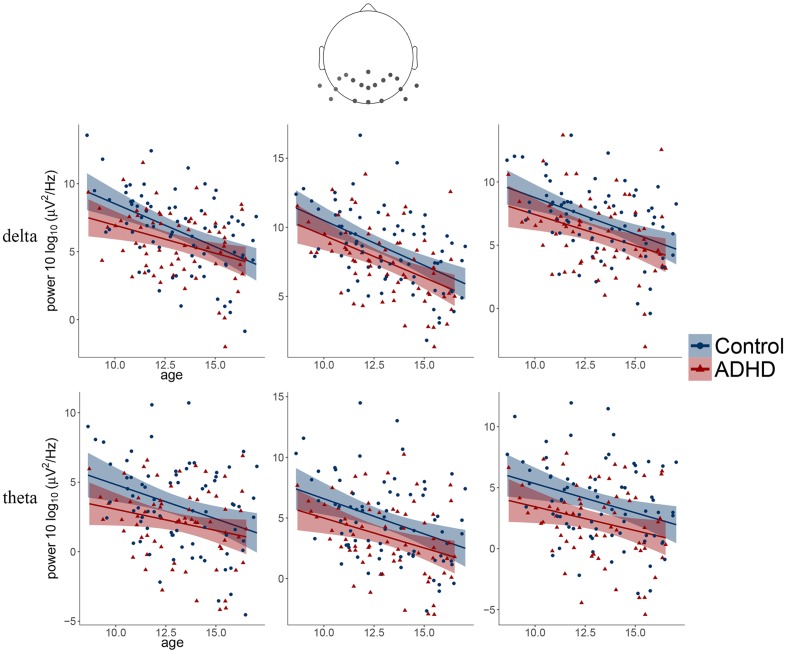
Scatter plots illustrating the main effects of age and diagnosis observed for delta and theta frequency bands over three posterior clusters (from left: left, middle, right).

The obtained results revealed a typical developmental effect of decreasing absolute EEG power with increasing age. Absolute EEG power was found to decrease linearly for delta, theta, and beta frequency bands across each of the scalp regions: anterior, central, and posterior in both groups (**Figure 2A**). Age-related changes in absolute power were much more pronounced in slow-wave frequencies, i.e., delta and theta (**Figure 3**), than in beta. The increase in the proportion of explained variance attributable to the effects of age varied across frequencies and clusters. For delta and theta Δ*R*^2^ was within 9.3–23.3% range, whereas for beta frequency it was more than five times smaller, ranging from 1.3 to 6.2%. The alpha absolute power was not significantly related to age.

Additionally, we observed the main effect of diagnosis, indicating differences between groups. The ADHD group had significantly lower absolute power in all frequency bands across centro-posterior clusters (**Figure 2B**), with the most pronounced difference in lower theta absolute power.

Additional analysis of the moderating effects of symptoms intensity on power spectra age-related changes in the ADHD group did not reveal any significant effects involving ADHD-RS scores in none of the models (all uncorrected *p*-values > 0.10).

## Discussion

The aim of this study was to investigate age-related changes in eyes-closed resting EEG activity in children and teenagers diagnosed with ADHD, in comparison to healthy controls. For that purpose, we included a large sample of participants across a broad age range and used regression analysis as a statistical tool. Such an approach allowed us to consider age as a continuous variable and obtain a more detailed view of the pace of the developmental changes in resting EEG in both studied groups. Our results did not reveal any age and ADHD interactions, suggesting that the rate of EEG power decrease is similar in ADHD and healthy participants across all regions and frequency bands. This pattern of results is in stark contrast with the delayed maturation hypothesis and suggests that age and presence of the disorder have an additive influence on resting EEG, which is congruent with the deviated brain activity maturation viewpoint.

We replicated the commonly reported EEG developmental pattern in healthy participants. Moreover, age-related changes in resting EEG activity were evident in both healthy and ADHD groups of children. EEG power underwent linear reductions with age, with the most prominent decreases of absolute power in the slow-wave frequencies, i.e., delta and theta, which is in line with the results of previous studies on healthy cohorts (Gibbs and Knott, 1949; Matousek and Petersen, 1973; Matsuura et al., 1985; Gasser et al., 1988; Somsen et al., 1997; Dustman et al., 1999; Boord et al., 2007). As aforementioned, the EEG activity developmental pattern has been linked to structural brain maturation expressed in significant gray matter tissue loss, possibly resulting from significant elimination of synapses (Huttenlocher and Dabholkar, 1997) and reduction in the neuropil (Purves, 1998; Whitford et al., 2007). These processes are thought to improve the efficiency of information processing, cognitive capacity, and executive functions (Kharitonova et al., 2013; Tamnes et al., 2013; Zhong et al., 2014). Neuroimaging studies have shown that the decrease in cortical gray matter thickness has substantial regional variation (Salat et al., 2005). Moreover, this development-related process is a combination of regionally specific cortical thinning in the cortical sulci and cortical thickening on the gyri (Vandekar et al., 2015). In pathologies, such as schizophrenia and ADHD, deficiencies of the expected cortical thinning during adolescence have been observed (Bakalar et al., 2009; Shaw et al., 2013). ADHD participants have been demonstrated to have a thinner cortex than controls in extensive areas of the brain, primarily the medial area (with the anterior cingulum included), superior prefrontal and precentral cortex (Shaw et al., 2006; Narr et al., 2009), and temporal lobe (Langevin et al., 2015) the surface of cortical areas was reported to be reduced in comparison to healthy participants as well (Shaw et al., 2012; for the review see, Kasparek et al., 2015). There is no direct evidence for the altered synaptic pruning that might be related to deficits in cortical thickness in ADHD. However, deficient pruning has been implicated in explanations of neuropsychiatric disorders, such as schizophrenia and autism (Penzes et al., 2011). Moreover, clinical, neuroimaging, and animal studies indicate that dopamine neurotransmission is dysregulated in ADHD. In a non-human primate model, the decrease in prefrontal pyramidal cell spine density associated with profound cognitive dysfunction has been shown to be caused by dopamine hyperstimulation (Tripp and Wickens, 2009; Genro et al., 2010).

As EEG activity is mainly driven by the synchronized synaptic activity at the cortex, a reduction in the number of synapses in the cortex has been proposed to lead to decreases in slow-wave delta and theta, but not high-frequencies of alpha or beta (Jenni and Carskadon, 2004; Whitford et al., 2007). The age in which the maximum dendritic spine density on pyramidal neurons occurs (Petanjek et al., 2011) correspond perfectly with the median age by which the peak thickness of the cortex is attained (Shaw et al., 2007), and a tight correlation between slow-wave activity and a variety of indices of cortical maturation derived from MR has been shown (Kurth et al., 2010; Buchmann et al., 2011). According to this view, the higher number of synapses during childhood could explain the high power of delta and theta oscillations. However, the process of synaptic pruning cannot be an explanation for the beta power decrease, since it presumably arises from asynchronous activity between pyramidal neurons (Whitford et al., 2007). On the other hand, the alpha frequency is thought to be driven by thalamic activity and therefore might be affected by structural changes in subcortical regions rather than cortical ones. This might explain the lack of the age-related decrease in alpha power in our study. Therefore, lower gray matter thickness implying a lower number of synapses in ADHD in comparison with healthy participants should result in lower delta and theta absolute power. However, contrarily, increased delta and theta power, mainly in the frontal regions, has been reported in ADHD in the vast majority of previous studies (for review see, Barry et al., 2003). We observed lower EEG power in all frequency bands in centro-posterior clusters in ADHD participants in comparison to healthy controls. The most prominent differences referred to decreased delta and theta in the ADHD group. This pattern of results, even though counterintuitive, is not unique in studies on the developmental changes in ADHD. For example, Shaw et al. (2011) reported age-related decrease in cortical thickness where, at a certain age, results of older controls match the gray matter thickness observed in younger ADHD children.

Nonetheless, our results, especially those for theta power, are in contrast to the previous results indicating elevated levels of theta power and theta/beta ratio (Chabot and Serfontein, 1996; Bresnahan et al., 1999; Koehler et al., 2009). However, more recent studies failed to replicate these effects (Loo et al., 2009; Ogrim et al., 2012; Liechti et al., 2013; Poil et al., 2014), showing no significant differences between ADHD and healthy participants in theta power. What is more, Liechti et al. (2013) noted the tendency of decreased relative theta and theta/beta ratio for ADHD, but the results did not reach significance. Instead, observed decreased power of alpha and beta was a commonly reported effect (for review see, Dykman et al., 1982; Barry et al., 2003; Clarke et al., 2008), also in the posterior regions (for review see, Lazzaro et al., 1998; Barry et al., 2003), and linked to cortical hypoarousal. Moreover, the high heterogeneity of ADHD in terms of EEG sub-types has been implicated by Clarke et al. (2011), who revealed five different groups on the basis of EEG characteristics. Two of the groups were characterized by decreased theta activity, which is similar to our observation. Patients in one of these groups were more likely to be aggressive than other ADHD children; patients of the other group presented autistic-like behaviors. ADHD is a highly comorbid disorder. More than 60% of ADHD patients exhibit one or more of the following co-existing disorders: 42–90% ODD and/or CD, 13–51% anxiety or depression, and 20–25% learning difficulties (Angold et al., 1999; Pliszka, 2000; Jensen et al., 2001; Bauermeister et al., 2007). Our ADHD group consisted of resident patients of one clinic, who were diagnosed according to both DSM-IV and ICD-10 diagnostic criteria using structuralized methods (Wolańczyk and Kołakowski, 2005) that involved interviews with parents and the observation of the patients’ behavior. Such an extensive process of diagnosis allowed us to include only children with a widely confirmed diagnosis. What is more, the distribution of comorbidities in our sample was close to those previously reported. That allows us to consider our groups as highly representative. It should be noted that most of the studies used different recruiting criteria, e.g., excluding children with comorbidities. This may be beneficial for the interpretation of results, however, such a group is not representative of the usual clinical outcomes.

It should be noted that Clarke et al. (2011) observed decreased theta power in the fronto-central regions, while we observed decreases in slow-waves absolute power in the centro-posterior clusters. The ADHD behavioral heterogeneity (subtypes and comorbid disorders) is likely linked with neurophysiological heterogeneity (diverse changes in brain morphology). Indeed, different studies reported different changes in various brain areas in ADHD (for review see, Kasparek et al., 2015) and this variability might be related to ADHD heterogeneity. Moreover, it is possible that regional variation in the development-related decrease in cortical gray matter thickness and regionally specific cortical thinning and thickening in sulci and gyri reported for healthy participants is not accurately followed in ADHD patients. Putatively, this is reflected in diverse EEG subtypes. Up to nine EEG subtypes have been proposed in children with and without ADHD (Chabot and Serfontein, 1996; Arns et al., 2008; Clarke et al., 2011; Johnstone et al., 2013) and this might explain differences between studies due to the uneven sampling of these subgroups.

Arns et al. (2013) conducted a meta-analysis on TBR in ADHD and found that heterogeneity among studies was very high. Studies varied on characteristics and factors such as study design, EEG technology, type of analysis, age of participants studied, sample size, medicating history, and whether or not participants diagnosed with co-morbidities were included. Moreover, in addition to differences between studies in characterizing EEG activity, studies of ADHD are also confounded by the high behavioral heterogeneity of this disorder.

The differences in our observations in comparison to previously published findings may also result from the application of a different methodology. First of all, we applied different statistical testing. We used regression analysis instead of comparing mean EEG power of each tested group with the others groups. We investigated a large group of children and teenagers in a wide age-range instead of comparing young participants to adults. Additionally, we applied different methods to analyze EEG activity. We used a different data cleaning approach, removing artifacts both manually and by excluding ICA components. The analysis was performed on clusters instead of testing the effects on single electrodes, which reduces the risk of interpreting noise rather than actual brain activity. The time of the recordings was also different; our recordings last for 5 min, and we applied a procedure is intended to mitigate drowsiness (eyes closed/eyes open). Most of the previous research adopted 20 min-long recordings.

Our analysis did not reveal any significant effects of interaction. Instead, we observed two main effects of development and group differences. The lack of interaction (a significant interaction would have provided evidence in favor of the developmental lag hypothesis) speaks for the presence of a permanent abnormality of EEG, at least within the examined time range. We observed the same pattern of age-related decreases in EEG power, particularly in slow wave activity, in both groups, whereas the difference between groups was stable in time; the EEG power of ADHD participants was always lower.

Taking into consideration the EEG-heterogeneity of ADHD and the structural differences reported in MRI studies, we assume that the lower EEG power may result from abnormally diminished gray matter volume in ADHD. Different trajectories of age-related decrease in cortical thickness in control and ADHD youths were reported where, at some point of time, results of older controls match the thickness observed in younger ADHD patients (Shaw et al., 2011). This might reflect two fundamentally different neurodevelopmental effects resulting in the observable changes in a single measured parameter. Nevertheless, there is a lack of the studies using simultaneous MRI/EEG recordings in ADHD, which would confirm our assumption.

Several alternatives to the explanations of decreasing EEG power with age based on reductions in cortical gray matter density have been proposed previously. For example, absolute metabolic rates were found to parallel gray matter maturation and maturational decrease in electrical activity (Feinberg et al., 1990; Boord et al., 2007; Feinberg and Campbell, 2010). Also, it has been shown that developmental global EEG low frequency power decrease is paralleled by global BOLD signal power decrease (Lüchinger et al., 2012). All of these global decreases in structural and metabolic markers, as well as global EEG and BOLD signal power decreases, indicate profound neuronal reorganization with age. Another explanation is that the spectral density of slow wave activity in resting EEG is largely determined by resonant thalamo-cortical loops (Llinás et al., 2005; Miskovic et al., 2015). Thalamic and thalamo-cortical loop maturation was implicated by EEG–MRI coupling and normalized BOLD power (Lüchinger et al., 2012). In this study performed in adults, adolescents and children it has been demonstrated that developmental EEG amplitude reductions match those found for the same age range with magnetoencephalography (MEG) (Lüchinger et al., 2012). The fact that MEG is insensitive to changes in physical skull properties (Puligheddu et al., 2005) corroborates neurodevelopmental nature of the observed effects. Therefore, we do not believe that the EEG effects in our study are strongly influenced by differences in physical properties of the head (e.g., skull thickness), which change during development. Based on the results obtained in the study with adult subjects it has been postulated that skull thickness may be neglected as a source of error in such cases (Hagemann et al., 2008). Moreover, the relationship between age and gamma revealed much stronger effect (Tierney et al., 2013) than an effect between skull thickness and resting EEG power (Hagemann et al., 2008). We cannot exclude, that developmental differences in skull thickness or other physical characteristics of the head could influence the age-related changes in EEG power spectra. However, according to Tierney et al. (2013) data, factors related to maturation explain more variance in EEG activity than differences in these physical factors do.

Several limitations of this study should be noted. Firstly, the cross-sectional character of this experiment restricts the possibility of obtaining a more dynamic overview of developmental changes in EEG activity. The younger group of subjects in our study might possibly be more heterogeneous than the older group, which may influence the results. Older ADHD children presented a persistent neurodevelopmental deficit in our study, whereas some of the younger participants might be expected to remit. Certainly, the cause and effect pathway could be established more reliably in a longitudinal study. There is a need to conduct longitudinal studies on EEG activity in healthy as well as clinical cohorts, which may provide valuable information on the dynamics of the alternations observed in ADHD and may have an impact on treatment approaches, e.g., stimulant and neurofeedback therapies. Secondly, the clinical group consisted of both combined and predominantly inattentive subtypes of ADHD. We found it highly representative of a typical ADHD population. The distribution of the ADHD subtypes and comorbidities in our sample was similar to that previously reported for ADHD cohorts. However, the “inattentive” subgroup was significantly smaller which did not allow us to investigate it in comparison to the “combined” and control groups. Also, the number of the female participants was too small to obtain valuable information on sex-differences.

## Conclusion

This study revealed that the resting-EEG developmental pattern was similar in ADHD and healthy controls. Even so, the ADHD group had consistently lower absolute EGG power, mostly in the theta frequency band. Our results are in line with deviant brain maturation hypothesis, as ADHD brain activity would not be considered the same as in healthy controls at any developmental stage, at least within the studied age range (9–16 years).

## Author Contributions

KG recruited control group of participants, carried out all of the study, performed the recordings, analyses, wrote the manuscript, prepared table and figures. MZ had substantial contribution to the analysis of the data and figures preparation. MB had substantial contribution to the statistical analyses of the data. ER-P recruited and diagnosed the ADHD participants. MK made comments and suggestions on the manuscript. AC-K had contribution to the conception of the work, interpretation of the data, and in writing the manuscript.

## Conflict of Interest Statement

The authors declare that the research was conducted in the absence of any commercial or financial relationships that could be construed as a potential conflict of interest.
